# Pneumomediastinum in COVID-19: a phenotype of severe COVID-19 pneumonitis? The results of the UK POETIC survey

**DOI:** 10.1183/13993003.02522-2021

**Published:** 2022-09-01

**Authors:** James Melhorn, Andrew Achaiah, Francesca M. Conway, Elizabeth M.F. Thompson, Erik W. Skyllberg, Joseph Durrant, Neda A. Hasan, Yasser Madani, Prasheena Naran, Bavithra Vijayakumar, Matthew J. Tate, Gareth E. Trevelyan, Irfan Zaki, Catherine A. Doig, Geraldine Lynch, Gill Warwick, Avinash Aujayeb, Karl A. Jackson, Hina Iftikhar, Jonathan H. Noble, Anthony Y.K.C. Ng, Mark Nugent, Philip J. Evans, Robert A. Hastings, Harry R. Bellenberg, Hannah Lawrence, Rachel L. Saville, Nikolas T. Johl, Adam N. Grey, Huw C. Ellis, Cheng Chen, Thomas L. Jones, Nadeem Maddekar, Shahul Leyakathali Khan, Ambreen Iqbal Muhammad, Hakim Ghani, Yadee Maung Maung Myint, Cecillia Rafique, Benjamin J. Pippard, Benjamin R.H. Irving, Fawad Ali, Viola H. Asimba, Aqeem Azam, Eleanor C. Barton, Malvika Bhatnagar, Matthew P. Blackburn, Kate J. Millington, Nicholas J. Budhram, Katherine L. Bunclark, Toshit P. Sapkal, Giles Dixon, Andrew J.E. Harries, Mohammad Ijaz, Vijayalakshmi Karunanithi, Samir Naik, Malik Aamaz Khan, Karishma Savlani, Vimal Kumar, Beatriz Lara Gallego, Noor A. Mahdi, Caitlin Morgan, Neena Patel, Elen W. Rowlands, Matthew S. Steward, Richard S. Thorley, Rebecca L. Wollerton, Sana Ullah, David M. Smith, Wojciech Lason, Anthony J. Rostron, Najib M. Rahman, Rob J. Hallifax

**Affiliations:** 1 Nuffield Dept of Medicine, John Radcliffe Hospital, University of Oxford, Oxford, UK; 2 National Institute for Health Research (NIHR) Oxford Biomedical Research Centre, Oxford, UK; 3 John Radcliffe Hospital, Oxford University Hospitals NHS Foundation Trust, Oxford, UK; 4 MRC Human Immunology Unit, Weatherall Institute of Molecular Medicine, University of Oxford, Oxford, UK; 5 Royal Brompton Hospital, National Heart and Lung Institute, London, UK; 6 St Bartholomew's Hospital, Barts Health NHS Trust, London, UK; 7 Newham University Hospital, Barts Health NHS Trust, London, UK; 8 Wexham Park Hospital, Frimley Health NHS Foundation Trust, Slough, UK; 9 Royal Free Hospital, Royal Free London NHS Foundation Trust, London, UK; 10 Chelsea and Westminster Hospital, National Heart and Lung Institute, Imperial College London, London, UK; 11 Queen Elizabeth University Hospital, NHS Greater Glasgow and Clyde, Glasgow, UK; 12 Royal Berkshire Hospital, Royal Berkshire NHS Foundation Trust, Reading, UK; 13 Southend University Hospital, Mid and South Essex NHS Foundation Trust, Southend, UK; 14 Prince of Wales Hospital, Cwm Taf Morgannwg University Health Board, Bridgend, UK; 15 The Royal Gwent Hospital, Aneurin Bevan Health Board, Newport, UK; 16 Northumbria Specialist Emergency Care Hospital, Northumbria Healthcare NHS Foundation Trust, Cramlington, UK; 17 Gloucester Royal Hospital, Gloucestershire Hospitals NHS Foundation Trust, Gloucester, UK; 18 Addenbrookes Hospital, Cambridge University Hospitals NHS Trust, Cambridge, UK; 19 Glangwilli General Hospital, Hywel Dda University Health Board, Carmarthen, UK; 20 Barnet General Hospital, Royal Free London NHS Foundation Trust, London, UK; 21 Royal Derby Hospital, University Hospitals of Derby and Burton NHS Foundation Trust, Derby, UK; 22 University Hospital of Wales, Cardiff and Vale University Health Board, Cardiff, UK; 23 Stoke Mandeville Hospital, Buckinghamshire Healthcare NHS Trust, Stoke Mandeville, UK; 24 Basingstoke and North Hampshire Hospital, Hampshire Hospitals NHS Foundation Trust, Basingstoke, UK; 25 Royal Stoke University Hospital, University Hospitals of North Midlands NHS Trust, Stoke, UK; 26 Watford General Hospital, West Hertfordshire Hospitals NHS Trust, Watford, UK; 27 University Hospital of North Tees, North Tees and Hartlepool NHS Foundation Trust, Hartlepool, UK; 28 Queen Alexandra Hospital, Portsmouth Hospitals University NHS Trust, Portsmouth, UK; 29 Bedford Hospital, Bedfordshire Hospitals NHS Foundation Trust, Bedford, UK; 30 Nottingham University Hospital, Nottingham University Hospitals NHS Trust, Nottingham, UK; 31 Royal Blackburn Teaching Hospital, East Lancashire Hospitals NHS Trust, Blackburn, UK; 32 The Grange University Hospital, Aneurin Bevan Health Board, Cwmbran, UK; 33 Darlington Memorial Hospital, County Durham and Darlington NHS Foundation Trust, Durham, UK; 34 Southport and Ormskirk District General Hospital, Southport and Ormskirk Hospital NHS Trust, Southport, UK; 35 Great Western Hospital, Great Western Hospital NHS Foundation Trust, Swindon, UK; 36 Norwich and Norfolk University Hospital, Norfolk and Norwich University Hospitals NHS Foundation Trust, Norwich, UK; 37 Royal United Hospitals Bath, The Royal United Hospitals Bath NHS Foundation Trust, Bath, UK; 38 Royal Glamorgan Hospital, Llantrisant, Cwm Taf University Health Board, Llantrisant, UK; 39 Wythenshawe Hospital, Manchester University NHS Foundation Trust, Manchester, UK; 40 The Princess Alexandra Hospital, The Princess Alexandra Hospital NHS Trust, Harlow, UK; 41 Luton and Dunstable University Hospital, Bedfordshire Hospitals NHS Foundation Trust, Luton, UK; 42 Kettering General Hospital, Kettering General Hospital NHS Foundation Trust, Kettering, UK; 43 University Hospital, University Hospitals Coventry and Warwickshire NHS Trust, Coventry, UK; 44 Lister Hospital, East and North Hertfordshire NHS Trust Stevenage, Stevenage, UK; 45 Musgrove Park Hospital, Somerset NHS Foundation Trust, Taunton, UK; 46 Whipps Cross Hospital, Barts Health NHS Trust, London, UK; 47 Neville Hall Hospital, Aneurin Bevan University Health Board, Abergavenny, UK; 48 Royal Devon and Exeter Hospital, Royal Devon and Exeter NHS Foundation Trust, Exeter, UK; 49 The Royal Cornwall Hospital, Royal Cornwall Hospitals NHS Trust, Truro, UK; 50 Ysbyty Glan Clwyd Hospital, Betsi Cadwaladr University Health Board, Rhyl, UK; 51 Integrated Critical Care Unit, Sunderland Royal Hospital, South Tyneside and Sunderland NHS Foundation Trust, Sunderland, UK; 52 Oxford Centre for Respiratory Medicine, Nuffield Dept of Medicine, University of Oxford, Oxford, UK

## Abstract

**Background:**

There is an emerging understanding that coronavirus disease 2019 (COVID-19) is associated with increased incidence of pneumomediastinum (PTM). We aimed to determine its incidence among patients hospitalised with COVID-19 in the UK and describe factors associated with outcome.

**Methods:**

A structured survey of PTM and its incidence was conducted from September 2020 to February 2021. UK-wide participation was solicited *via* respiratory research networks. Identified patients had severe acute respiratory syndrome coronavirus 2 (SARS-CoV-2) infection and radiologically proven PTM. The primary outcomes were to determine incidence of PTM in COVID-19 and to investigate risk factors associated with patient mortality.

**Results:**

377 cases of PTM in COVID-19 were identified from 58 484 inpatients with COVID-19 at 53 hospitals during the study period, giving an incidence of 0.64%. Overall 120-day mortality in COVID-19 PTM was 195 out of 377 (51.7%). PTM in COVID-19 was associated with high rates of mechanical ventilation. 172 out of 377 patients (45.6%) were mechanically ventilated at the point of diagnosis. Mechanical ventilation was the most important predictor of mortality in COVID-19 PTM at the time of diagnosis and thereafter (p<0.001), along with increasing age (p<0.01) and diabetes mellitus (p=0.08). Switching patients from continuous positive airway pressure support to oxygen or high-flow nasal oxygen after the diagnosis of PTM was not associated with difference in mortality.

**Conclusions:**

PTM appears to be a marker of severe COVID-19 pneumonitis. The majority of patients in whom PTM was identified had not been mechanically ventilated at the point of diagnosis.

## Abstract 2

**Roughly 0.6% of patients admitted with COVID-19 have pneumomediastinum identified. The finding is associated with severe COVID-19 and high mortality.** https://bit.ly/3nXlaR5

## Introduction

Pneumomediastinum (PTM) is the abnormal presence of air or gas in the mediastinum. Spontaneous PTM is rare, appearing in approximately 1 in 33 000 hospital admissions [1]. PTM has a higher reported incidence among patients receiving positive pressure ventilation (PPV), particularly those with acute respiratory distress syndrome (ARDS) [2, 3].

The coronavirus disease 2019 (COVID-19) pandemic has seen a remarkable increase in the number of patients receiving PPV within a given period, with many patients with COVID-19 pneumonitis meeting ARDS criteria [4, 5]. The publication of several case reports and small series of PTM in patients with COVID-19 could be viewed in this context [5–8]. There have, however, been a number of reports of PTM occurring in COVID-19 pneumonitis without PPV [9–11]. The true incidence of PTM in COVID-19 and its relationship to PPV remains unclear. In addition, whether management should be altered after the identification of PTM is not known.

We report a multicentre observational study (POETIC) of 377 cases of COVID-19 PTM from 53 hospitals in the UK between September 2020 and February 2021. We describe the incidence and risk factors associated with PTM in COVID-19 and associations with mortality.

## Materials and methods

### Study population

The POETIC study recruited across the UK. It was advertised *via* national and regional trainee research networks including the Pulmonary Research Inter-Site Matrix (PRISM) and North West Collaborative Respiratory Research (NCORR).

Participating institutions contributed cases of PTM in inpatients with COVID-19 identified between 1 September 2020 and 31 January 2021 (supplementary table S2). The diagnosis of PTM was based on computed tomography (CT) or plain radiography of the chest, and the diagnosis of COVID-19 was based on a positive severe acute respiratory syndrome coronavirus 2 (SARS-CoV-2) PCR result or evidence of COVID-19 pneumonitis on CT imaging and a clear clinical history. All participating institutions searched radiology reports using the key words “pneumomediastinum”, “pneumothorax” or “subcutaneous emphysema”, together with patient lists from medical and respiratory wards and intensive care units to ensure all cases were identified. Anonymised data were collected for each case. These included demographics, past medical history, radiological findings, clinical outcomes, and respiratory settings from all respiratory support prior to and after the diagnosis of PTM. Follow-up at ≥120 days was recorded for all patients and all cases of interhospital transfer were cross-checked to ensure no duplication. In order to accurately estimate incidence, data were collected on the total numbers of patients admitted during this period who were SARS-CoV-2-positive on PCR testing and the proportion who underwent chest CT imaging at each institution.

All data pertaining to inspiratory oxygen fraction (*F*_IO_2__) were normalised to a uniform scale prior to analyses (*e.g.* all patients receiving 15 L of oxygen *via* a nonrebreather mask with reservoir were assigned an *F*_IO_2__ of 90%). A variety of devices were used to deliver high-flow nasal oxygen (HFNO) and continuous positive airway pressure (CPAP). Positive end-expiratory pressure (PEEP) and *F*_IO_2__ for patients receiving CPAP were normalised to values based on data from the Association of Respiratory Technology and Physiology (www.artp.org.uk/COVID19). PEEP for patients on HFNO was estimated and normalised based on published physiological data from Groves and Tobin [12]. Full details can be found in supplementary table S3a and b and supplementary figure S3c.

Ethical approval was obtained from the Oxford University Hospitals NHS Foundation Trust (UK) audit committee with additional ethical approval from local NHS Trusts where relevant. All data were collected retrospectively and entered by local physicians in anonymised fashion without linkage to patient identifiers.

### Statistical methods

Patient data are presented as frequency (percentage) for categorical data and mean with standard deviation for continuous data. Where data were not normally distributed the median (interquartile range (IQR)) is presented. Differences in categorical data are presented using the Chi-squared test. For comparisons of continuous normally distributed data, two-sided independent t-tests were used. All outcomes quoted are outcomes at 120 days. Variable entry into regression models was performed backwards stepwise. Analyses were performed using SPSS version 28 (IBM, Armonk, NY, USA). Figures were created in R version 4.0.3 (www.r-project.org). We present the article in accordance with the STROBE (Strengthening the Reporting of Observational studies in Epidemiology) checklist.

## Results

A total of 377 cases of PTM were detected of whom 98.4% had a positive SARS-CoV-2 PCR and the remainder were diagnosed clinically. The diagnosis of PTM was made or confirmed on chest CT scan in 318 cases (84.4%). For 147 out of 318 (46.2%) of the cases diagnosed by CT scan, PTM had not been visible on a preceding chest radiograph. Outcome data were obtained for all patients and incidence data from all included hospitals. All other data were ≥95% complete for all parameters.

### Incidence

There were 58 484 PCR-positive inpatient admissions for the period 1 September 2020 to 31 January 2021 within the 53 participating hospitals (mean±sd 1103±611 COVID-19 inpatients per hospital). The incidence of PTM was 0.64% (95% CI 0.58–0.71%) per COVID-19 inpatient admission with a mean±sd number of PTM cases of 7.1±4.8 per hospital. 12 703 of the 58 484 PCR-positive inpatients (21.7%) underwent chest CT imaging during admission with a mean±sd number of CT scans performed per hospital of 240±200. The relationship between the number of total inpatient admissions, use of chest CT imaging and number of cases of PTM across hospitals is presented in supplementary figure S4.

### Demographics

The median (IQR) age was 60 (52–78) years. Male patients were over-represented with 277 (73.5%) male to 100 (26.5%) female patients. The most prevalent medical comorbidities were hypertension (32.4%), diabetes mellitus (21.5%), asthma (19.4%), obesity (10.6%), ischaemic heart disease/left ventricular systolic dysfunction (8.8%) and chronic kidney disease (4.5%). Three patients were pregnant. The median (IQR) duration of symptoms prior to admission to hospital was 7 (5–10) days while the median (IQR) duration from admission to the identification of PTM on imaging was 7 (5–12.3) days. Chest pain was a feature of the presenting complaint in 11.9% of patients.

315 out of 377 (83.6%) patients were considered eligible for mechanical ventilation should it be required. Eligibility for mechanical ventilation was a clinical decision recorded in the notes. Treatment of the remaining 62 patients was limited to CPAP support should it be required. Given the differences in management between these two groups they are considered separately in our regression analyses examining factors linked to patient outcome.

### Management

241 out of 315 (76.5%) patients considered eligible for escalation to mechanical ventilation were mechanically ventilated at some point during their admission. 172 of these 241 (71.4%) patients were mechanically ventilated prior to the diagnosis of PTM. PTM was detected within 24 h following intubation in 38 out of 241 (15.8%) of these patients. 24 out of 241 (10.0%) of these patients went on to receive extracorporeal membrane oxygenation (ECMO). Of the 377 patients eligible to receive CPAP, 256 out of 377 (67.9%) had received CPAP prior to the diagnosis of PTM. Conscious noninvasive bilevel positive airway pressure (BiPAP) ventilation was used at some point in the admissions of nine out of 377 (2.4%) patients, other than its use in weaning patients from mechanical ventilation. Four of these nine patients were in type 2 respiratory failure. The indication for use of BiPAP for the other five patients was not clear. Given the few patients who received BiPAP and the variation in its use, we have excluded it from our analyses.

**FIGURE 1:**
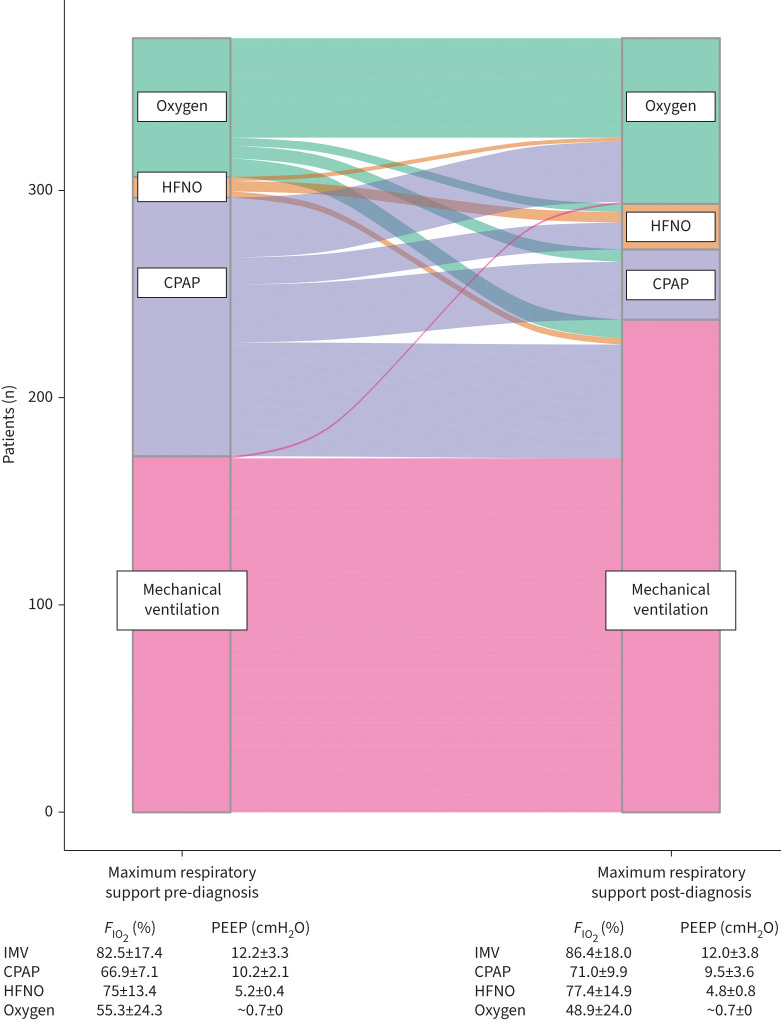
Sankey plot charting the maximum respiratory support given to all patients 4 h before the diagnosis of pneumomediastinum (PTM) and then following the diagnosis of PTM (n=374). The mean±sd inspiratory oxygen fraction (*F*_IO_2__) and positive end-expiratory pressure (PEEP) received on these levels of support is given in the tables below. HFNO: high-flow nasal oxygen; CPAP: continuous positive airway pressure; IMV: invasive mechanical ventilation.

The maximum respiratory support provided to all patients before and after the diagnosis of PTM is described in figure 1. Four patients whose treatment was limited to noninvasive respiratory support were switched from CPAP to oxygen at the point of diagnosis of PTM as part of a decision to initiate palliative treatment. Two patients were managed on room air throughout.

**FIGURE 2:**
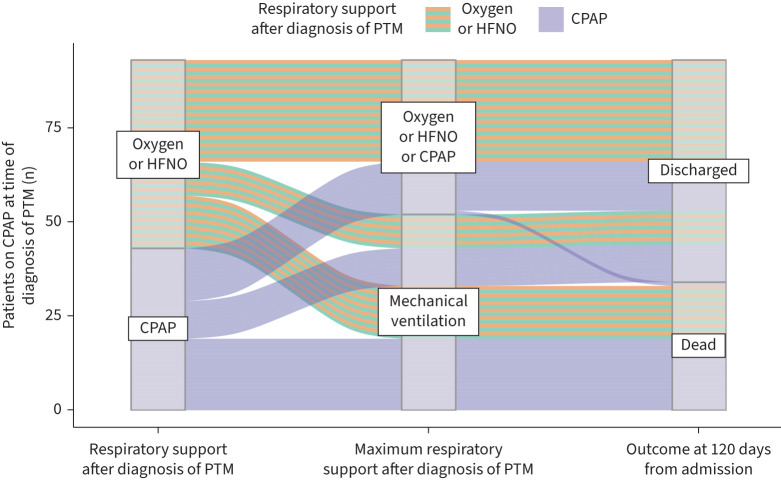
Alluvial plot describing the trajectory of 93 patients eligible for mechanical ventilation who had been on continuous positive airway pressure (CPAP) at the point of diagnosis of pneumomediastinum (PTM). At the point of diagnosis there was no statistical difference in age, maximum inspiratory oxygen fraction or maximum positive end-expiratory pressure received between those patients subsequently maintained on CPAP and those subsequently switched to oxygen or high-flow nasal oxygen (HFNO).

Alteration of respiratory support at the time of diagnosis is illustrated in figure 1. Most patients whose respiratory support was changed after the diagnosis of PTM were on CPAP. At the point of diagnosis of PTM, 93 patients eligible for mechanical ventilation were on CPAP. 50 (53.8%) of these patients were switched immediately on diagnosis of PTM to either oxygen or HFNO therapy, creating two subgroups amenable to analysis: the 50 switched to oxygen or HFNO and the 43 continuing on CPAP. These two subgroups were retrospectively well matched at the point of diagnosis by age (CPAP mean age 57.0 years *versus* oxygen or HFNO 55.6 years; p=0.51), by the maximum *F*_IO_2__ they had received (CPAP mean *F*_IO_2__ 66% *versus* oxygen or HFNO 68%; p=0.15) or by the maximum PEEP they had received (CPAP mean PEEP 10.4 cmH_2_O *versus* oxygen or HFNO 9.8 cmH_2_O; p=0.19). The subsequent trajectory of these two subgroups is illustrated in figure 2. Associations of change in mode of respiratory support and mortality for these patients were examined by ANOVA. There was no significant main effect of switching support from CPAP to oxygen or HFNO on outcome. There was, however, a main effect of mechanical ventilation as a factor associated with mortality for both subgroups (p<0.001).

### Co-occurrence of pneumothorax, subcutaneous emphysema and complications associated with PTM

**FIGURE 3:**
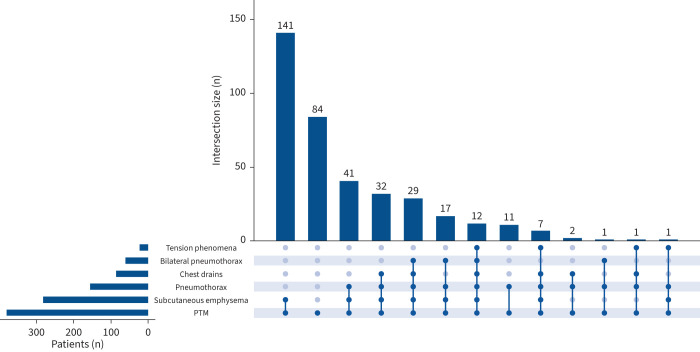
UpSet plot illustrating the co-occurrence of pneumomediastinum (PTM) with subcutaneous emphysema, pneumothorax and tension phenomena and the use of intercostal chest drains (n=377). Intersection size is the number of patients with only those features indicated. Bilateral pneumothorax was ascribed to pneumothoraces occurring on both sides of the thorax within the same admission.

Pneumothorax was seen concurrently in 154 out of 377 (40.8%) patients of whom 139 out of 154 (90.1%) also had subcutaneous emphysema. Subcutaneous emphysema was seen in a total of 280 out of the 377 (74.3%) patients. The co-occurrence of PTM with pneumothorax, subcutaneous emphysema and tension phenomena and the use of intercostal drains are displayed in figure 3. The numbers and frequencies of intercostal chest drains inserted are presented in supplementary figure S5. In cases associated with subcutaneous emphysema, subcutaneous drains were employed in six (1.6%) cases. In five of these six cases subcutaneous drains were inserted for threatened or actual tension subcutaneous emphysema. There were four (1.1%) instances of mediastinal drains being used. In one of these four cases the mediastinal drain was inserted as an emergency bedside procedure for suspected tension PTM and tension subcutaneous emphysema. In the other three out of four cases the mediastinal drain was inserted to obviate possible tension PTM. These four mediastinal drains were inserted in patients at four different hospitals, each without on-site cardiothoracic services. There were 14 cases of tension pneumothorax. During 10 cases of suspected tension phenomena, bilateral intercostal drains were inserted as an emergency procedure. Seven of these 10 cases were performed without prior radiographic evidence of pneumothorax.

The development of pneumothorax was not associated with increased risk of death for our cohort (table 1), including the subset of 16 patients who were mechanically ventilated before pneumothorax developed (11 out of 116 (9.5%) were among those patients who subsequently died, while five out of 56 (8.9%) were among those discharged; p=0.9). There were two cases of pneumoperitoneum. Both of these cases were in mechanically ventilated patients.

**TABLE 1:** Univariate analyses: factors of the presentation and their association with outcome at 120 days (n=377)

	**Outcome**		**OR (95% CI) for death**	**p-value**
	**Dead**	**Discharged**		
**Mechanically ventilated at any time (of those eligible) (n=315)**	160/161 (99.8)	81/154 (52.6)	144.2 (19.7–1056)	<0.001
**Respiratory support at time of diagnosis (n=375)**				
Oxygen	24/199 (12.1)	44/176 (25.0)	Reference	
HFNO	4/199 (2.0)	6/176 (3.4)	1.2 (0.3–4.8)	0.77
CPAP	55/199 (27.6)	70/176 (39.8)	1.4 (0.8–2.7)	0.24
Mechanical ventilation	116/199 (58.3)	56/176 (31.8)	3.8 (2.1–6.9)	<0.001
**Age (years)**	62.1±11.4	55.8±13.1		<0.001
**Subcutaneous emphysema**	167/200 (83.5)	113/177 (63.8)	2.9 (1.8–4.6)	<0.001
**Ischaemic heart disease or left ventricular systolic dysfunction**	26/200 (13.0)	7/177 (4.0)	3.6 (1.5–8.6)	<0.01
**Hypertension**	76/200 (38.0)	46/177 (26.0)	1.9 (1.2–2.9)	0.01
**Diabetes mellitus**	53/200 (26.5)	28/177 (15.8)	1.9 (1.2–3.2)	0.01
**Radiographic progression of pneumomediastinum**	58/200 (29.0)	31/177 (17.5)	1.9 (1.2–3.2)	0.01
**ECMO (of those eligible) (n=241)**	11/160 (6.9)	13/81 (16.0)	0.4 (0.2–0.9)	0.03
**Male**	140/200 (70.0)	137/177 (77.4)	0.7 (0.4–1.1)	0.11
**Chest pain at presentation (n=365)**	19/193 (9.8)	26/172 (15.1)	0.6 (0.3–1.2)	0.13
**Chronic kidney disease**	11/200 (6.0)	5/177 (2.8)	1.7 (0.6–4.6)	0.21
**Asthma**	43/200 (21.5)	30/177 (16.9)	1.3 (0.8–2.3)	0.27
**Tension phenomena**	13/200 (6.5)	7/177 (4.0)	1.7 (0.7–4.3)	0.28
**Obesity (BMI ≥35 kg·m^−2^)**	23/200 (11.5)	17/177 (9.6)	1.2 (0.6–0.4)	0.55
**Pneumothorax (at anytime)**	84/200 (42.0)	70/177 (39.5)	1.1 (0.7–4.7)	0.63

Data are presented as n/N (%) or mean±sd, unless otherwise stated. HFNO: high-flow nasal oxygen; CPAP: continuous positive airway pressure; ECMO: extracorporeal membrane oxygenation; BMI: body mass index.

In eight cases PTM appeared following an interventional procedure that could potentially represent a separate mechanism for occurrence, *e.g.* tracheostomy, and these cases are included in the final analysis. Analyses were performed excluding these cases without any statistically significant deviation from the results presented.

### Mortality

At 120 days from admission, 175 out of 377 (46.4%) patients had been discharged and 195 out of 377 (51.7%) patients had died. Of the seven patients still in hospital at 120 days, at time of writing one patient had died on day 162 of their admission. Three patients remained mechanically ventilated on days 131, 146 and 150 of admission. One patient had been extubated but remained within intensive care on day 132 of admission. These five patients were categorised with those who had died at 120 days in all outcome analyses. The remaining two patients were medically fit for discharge to rehabilitation facilities at days 137 and 149 of admission. They were categorised with patients discharged at 120 days in all outcome analyses. A breakdown of mortality is provided in supplementary table S6a and b according to whether patients were eligible for mechanical ventilation or limited to CPAP support.

Factors of the presentation and association with outcome are presented for all patients in table 1. All factors significantly associated with mortality in univariate analyses were entered into binary regression prediction models with the exception of the use of ECMO, which was excluded as the direction of association for this variable was in favour of discharge rather than death. The variable “radiographic progression of PTM” was excluded where the model was conducted from the point of diagnosis.

A regression model comparing the predictive utility of variables for mortality at 120 days from the point of diagnosis for patients eligible for all treatment is presented in table 2. Further models looking at the predictive utility of the same variables for mortality across the duration of hospital admission are presented for patients eligible for all treatment in supplementary table S7 and for those limited to CPAP in supplementary table S8.

**TABLE 2:** Binary logistic regression model of factors predictive of death at 120 days from the point of diagnosis of pneumomediastinum (all patients eligible for mechanical ventilation, n=315)

	**B±se**	**OR or % increase per unit (95% CI)**	**p-value**
**Mechanically ventilated (at diagnosis)**	1.40±0.26	4.0 (2.4–6.7)	<0.001
**Age**	−0.38±0.12	3.7% (1.4–5.9%) per year	<0.01
**Diabetes mellitus**	0.59±0.34	1.8 (0.9–3.5)	0.08

All variables significantly associated with mortality in univariate analyses were entered into the model backwards stepwise. The model produces a prediction accuracy for outcome of 68.4% *versus* a 51.1% default accuracy. Model R^2^=0.251, Nagelkerke, χ^2^(6)=65.2, p<0.001. Constant B±se=2.38±0.79. Variables in the regression but not listed: subcutaneous emphysema (p=0.12), hypertension (p=0.35) and ischaemic heart disease/left ventricular systolic dysfunction (p=0.50).

## Discussion

These data comprise the largest series of PTM in COVID-19 to date. In comparison with other series we sought to accurately represent the incidence of PTM in COVID-19 during the period of the survey, *i.e.* the UK's “second wave” of the pandemic. Hospital records and radiology reports were systematically reviewed in each centre. Hospitals that did not observe cases of PTM but provided accurate incidence data were included. However, hospital participation was sought *via* trainee research networks and this may have resulted in inclusion bias.

Our estimate of incidence is also subject to diagnostic biases. We identified cases through radiology reports, which may not always reference a relevant finding. The main mode of diagnosis of PTM was CT imaging and there was considerable variation in the use of CT by participating hospitals (supplementary figure S4). Many chest CT scans were pulmonary angiogram studies assaying for pulmonary emboli, not for PTM. For 46.2% of the patients diagnosed with PTM on thoracic CT the PTM was not visible on their preceding chest radiograph. As only 21.7% of our total denominator population of 58 484 COVID-19 PCR-positive inpatients had thoracic CT imaging performed during their admissions, there are likely to be a number of undetected cases of PTM in our denominator population. These unknown cases may have had a more benign disease trajectory than the cases identified.

With these caveats, these data demonstrate an incidence of PTM in COVID-19 of 0.64% per inpatient admission and 3.0% per COVID-19 inpatients undergoing thoracic CT. This incidence is similar to rates reported by two other studies of PTM in hospitalised COVID-19 populations from Brazil and Romania, of 0.51% and 0.67%, respectively [13, 14]. The incidence of “spontaneous” PTM in COVID-19 in this cohort, *i.e.* without any PPV *via* mechanical ventilation or CPAP, was 77 out of 58 484 (0.13%). This is much higher than estimated background rates of non-COVID-19 “spontaneous” PTM. The largest study of non-COVID-19 “spontaneous” PTM in the literature with a defined denominator population identified 41 cases of PTM from 1 824 967 emergency department admissions over 16 years (0.00002%) [15].

The mean age of the cohort (59.1 years) is consistent with inpatient international COVID-19 PTM cohorts from Brazil, Romania, Turkey, Pakistan and the USA [13, 16–19]. It is somewhat younger than the mean age of general COVID-19 inpatients in the UK, according to the largest epidemiological study (70.4 years) [20]. There could be pathophysiological reasons why COVID-19 inpatients who develop PTM are younger than the hospital population average (we note that background rates of non-COVID-19 PTM typically occur in younger adults) [1, 14, 15]. It could reflect bias towards more frequent imaging in younger patients who are usually eligible for all treatments, with an artificial reduction in the identification of PTM in older patient groups. A younger mean age is also representative of trends in patients hospitalised with COVID-19 during the “second wave” in the UK [21].

Pneumothorax was found to coexist with PTM in 40.3% of cases. This compares to reported rates of between 20.0% and 72.7% in other series with more than 10 patients [6, 13, 16–18, 22]. There was no finding of an effect on mortality of pneumothorax within this cohort nor specifically for those patients who were mechanically ventilated when pneumothorax occurred. This contrasts with the findings of Marciniak *et al.* [23], who report an increased risk of mortality with COVID-19 pneumothorax in a large dataset of UK inpatients, and Chopra *et al.* [24], who found increased mortality in mechanically ventilated patients across four intensive care units in the USA. As concurrent PTM was not reported in the Marciniak *et al.* [23] study and was relevant to 30% of the patients in the Chopra *et al.* [24] study, it is not clear how comparable these patient groups are to our cohort. The extent to which pneumothorax and PTM are manifestations of barotrauma in COVID-19 underwritten by a pathophysiological process and the extent to which they are distinct entities remains to be determined.

Subcutaneous emphysema was seen in 77.9% of COVID-19 PTM patients (see CT imaging in supplementary figure S9). Subcutaneous emphysema has been documented at rates of between 63.6% and 90.5% in other COVID-19 PTM series with more than 10 patients [13, 16, 17]. This result is in keeping with high reported rates of subcutaneous emphysema in spontaneous non-COVID PTM of up to 100% [1], and in excess of lower rates of co-occurrence between subcutaneous emphysema and non-COVID-19 pneumothorax of up to 20% [25]. It would suggest that subcutaneous emphysema is a feature strongly associated with PTM and not specifically to COVID-19 PTM. It is acknowledged, however, that co-occurrence of subcutaneous emphysema and PTM may be subject to diagnostic bias, with patients presenting with subcutaneous emphysema more likely to have CT imaging and subsequent revealing of a diagnosis of PTM.

It is not possible to determine the effect of different ventilatory strategies on outcome within an observational study such as this. However, we examined this for those patients eligible for mechanical ventilation who were on CPAP when PTM was diagnosed. The role of CPAP in patients with PTM is a clinically important question: analysis of changes in respiratory support after diagnosis of PTM permits an exploration of physician preferences regarding respiratory support, and by inference use of PEEP, in PTM. Those patients who remained on CPAP immediately after diagnosis of PTM were retrospectively well matched with those patients who were switched immediately to oxygen or HFNO by age, maximum *F*_IO_2__ and maximum PEEP. There was no difference in survival at 120 days between these subgroups. Thus, the current data do not support a policy of taking patients off CPAP when PTM is diagnosed, although we acknowledge potential confounders.

The 120-day mortality rate for patients with COVID-19 PTM of 51.7% is in keeping with reported mortality rates of 47.7–72.2% in other COVID-19 PTM cohorts [13, 16, 17]. The severity of COVID-19 illness is demonstrated by the high mean levels of *F*_IO_2__ and PEEP before and after the diagnosis of PTM was made (figure 1). Only two patients (0.5%) were managed on room air throughout admission. The number of patients who were mechanically ventilated at some point during their admission was remarkable at 76.5% of those eligible compared with the UK average for mechanical ventilation of COVID-19 inpatients of 8.8% [20]. Mechanical ventilation was unsurprisingly an important prognostic factor and dominant variable in outcome prediction models (table 2). It is a ubiquitous event in the trajectory of a deteriorating patient eligible for this support. Only one eligible patient in our cohort died without having been mechanical ventilated.

High rates of mechanical ventilation in COVID-19 PTM have been reported in other general hospital inpatient COVID-19 studies [13, 16]. This may reflect a confounding relationship between more severe illness and higher rates of CT scanning and detection in high-care environments. It may also indicate an important role for mechanical ventilation in the development of PTM in COVID-19. However, the majority of this cohort, 205 out of 377 patients (54.4%), were not mechanically ventilated at the point the diagnosis of PTM was made. Mechanical ventilation was therefore not a sufficient or necessary mechanism of PTM for the majority of patients.

Different mechanisms of PTM are described in the literature, including posterior membrane tracheal lesion or rupture due to coughing [26]. The “Macklin effect” describes PTM secondary to the rupture of marginal alveoli due to a steeply increased pressure gradient between the alveolus and the interstitial space [27]. After rupture of the alveolus, air dissects centripetally along the sheaths of the broncho-vascular bundles into the mediastinum. Depending on volume and pressure, air can be decompressed along cervical fascial planes into the subcutaneous tissues of the chest wall, neck or face. Air may rupture the relatively thin mediastinal pleura to enter the pleural space causing unilateral or bilateral pneumothorax and/or pneumopericardium/pneumoperitoneum. Macklin and Macklin [27] believed the effect could be benign or result in circulatory collapse if air directly compressed the pericardium or venous return: a tension PTM or pneumothorax. Air in the broncho-vascular bundles could also have a pernicious splinting effect leading to hyperinflation and low compliance with vascular compression and poor gas exchange, “malignant interstitial emphysema”. The “Macklin effect” was inspired by physician descriptions of “pulmonary interstitial emphysema” in patients suffering severe respiratory illness during the 1918–1920 influenza pandemic [28, 29], the pathophysiology of which may bear comparison with the COVID-19 pandemic.

The “Macklin effect” offers a plausible mechanism for PTM in COVID-19, whereby the pneumonitis creates an altered diathesis for the rupture of alveoli and the emergence of PTM. The proposition that COVID-19 PTM patients have severe pneumonitis is supported by cohort studies that describe high radiological scores of pneumonitis in COVID-19 PTM [10, 16–18]. The complimentary findings in our cohort of high levels of respiratory support (taken to represent severe pneumonitis), high rates of subcutaneous emphysema, episodes of tension phenomena and low rates of chest pain (compared with spontaneous PTM) support the “Macklin effect” as the likely mechanism of PTM in COVID-19. The previous 2002–2004 SARS epidemic also saw an increase in case reports of PTM [30] and this may reflect similar pathophysiology.

Future studies among mechanically ventilated patients with COVID-19 may elucidate whether strategies which modify trans-alveolar pressure have any association with development or progression of PTM. We notice that the 40 out of 377 (10.6%) patients in our cohort with obesity were not at increased risk of death compared with other patients with PTM and speculate whether this could relate to mass loading around the chest wall and/or abdomen with reduction of alveolar compliance and/or trans-alveolar gradients. Propensity-matched cohort analyses may address whether the development of PTM confers increased mortality risk, beyond severe pneumonitis, or whether development of PTM is affected by disease-modifying drugs such as dexamethasone (standard of care for our cohort) or different variants of SARS-CoV-2.

In summary, this study is the largest reported series of PTM in COVID-19. PTM appears to be a marker of severe pneumonitis and not necessarily as a result of the use of PPV. There was no evidence of increased harm by continuing CPAP in COVID-19 patients who developed PTM.

## Supplementary material

10.1183/13993003.02522-2021.Supp1 **Please note:** supplementary material is not edited by the Editorial Office, and is uploaded as it has been supplied by the author.
Supplementary material ERJ-02522-2021.Supplement

## Shareable PDF

10.1183/13993003.02522-2021.Shareable1 This one-page PDF can be shared freely online.
Shareable PDF ERJ-02522-2021.Shareable
