# Supplementary material for: Pneumomediastinum in COVID-19: a phenotype of severe COVID-19 pneumonitis? The results of the UK POETIC survey
Source: Eur Respir J. 2022 Sep 1;60(3):2102522. doi: 10.1183/13993003.02522-2021 (PMC8832377; doi:10.1183/13993003.02522-2021)
Supplement: Supplementary file 1 [file ERJ-02522-2021.Supplement.pdf]

Pneumomediastinum in COVID-19: a phenotype of severe COVID-19 pneumonitis? The results of the United Kingdom (POETIC) survey

Online Data Supplement
(S1 – S9)

James Melhorn, Andrew Achaiah, Francesca M. Conway, Elizabeth M. F. Thompson, Erik W. Skyllberg, Joseph Durrant, Neda A. Hasan, Yasser Madani, Prasheena Naran, Bavithra Vijayakumar, Matthew J. Tate, Gareth E. Trevelyan, Irfan Zaki, Catherine A. Doig, Geraldine Lynch, Gill Warwick , Avinash Aujayeb , Karl A. Jackson, Hina Iftikhar, Jonathan H. Noble, Anthony Y. K. C. Ng, Mark Nugent, Philip J. Evans, Robert A. Hastings, Harry R. Bellenberg, Hannah Lawrence, Rachel L. Saville, Nikolas T. Johl, Adam N. Grey, Huw C. Ellis, Cheng Chen, Thomas L. Jones, Nadeem Maddekar, Shahul Leyakathali Khan, Ambreen Iqbal Muhammad, Hakim Ghani, Yadee Maung Maung Myint, Cecillia Rafique, Benjamin J. Pippard, Benjamin R. H. Irving, Fawad Ali, Viola H. Asimba, Aqeem Azam, Eleanor C. Barton, Malvika Bhatnagar, Matthew P. Blackburn, Kate J. Millington, Nicholas J. Budhram, Katherine L. Bunclark, Toshit P. Sapkal, Giles Dixon, Andrew J. E. Harries, Mohammad Ijaz, Vijayalakshmi Karunanithi, Samir Naik, Malik Aamaz Khan, Karishma Savlani, Vimal Kumar, Beatriz Lara Gallego, Noor A. Mahdi, Caitlin Morgan, Neena Patel, Elen W. Rowlands, Matthew S. Steward, Richard S. Thorley, Rebecca L. Wollerton, Sana Ullah, David M. Smith, Wojciech Lason, Anthony J Rostron, Najib M Rahman and Rob J Hallifax

Glossary of terms used

ARDS = acute respiratory distress syndrome
BiPaP = conscious non-invasive bi-level positive airways pressure
COVID-19 = coronavirus 2019 infection
CPAP = continuous positive airways pressure
CT = computed tomography
CXR = chest radiograph
ECMO = extracorporeal membrane oxygenation
FiO2 = fraction of inspired oxygen
IHD = ischaemic heart disease
HFNO = high flow nasal oxygen
LVSD = left ventricular systolic dysfunction
MV = invasively mechanically ventilated
PEEP = positive end expiratory pressure
PPV = positive pressure ventilation
PTM = pneumomediastinum
UK = United Kingdom

S1

Prior to performing the current study we conducted a literature search of evidence on the subject. We searched MEDLINE and PubMed for original peer-reviewed cohort studies describing the incidence of pneumomediastinum in COVID-19 between March 2020 and June 2021. Search terms were “Pneumomediastinum” AND “COVID-19” OR “Barotrauma” AND “COVID-19” OR “Pneumothorax” AND “COVID-19”. Only reports published in English that included at least 5 cases and with estimates of a background population were included. Our search yielded 15 studies. These are detailed in table S1 below

Table S1. Previously published cohort studies with ≥5 cases of pneumomediastinum (PTM) and an identified denominator population.

**Study and country of origin**	**Single or Multicentre**	**Date Published**	**Number of cases of PTM**	**% cases diagnosed by thoracic CT**	**PTM with concurrent PTX**	**PTM with concurrent SCE**	**Number of denominator population**	**Denominator patient population**	**% cases in denominator PCR positive**
McGuiness et al (USA)[1]	Single	Nov 2020	59	CXR diagnoses	Not stated	Not stated	601	Mechanically Ventilated	100%
Kangas-Dick et al[2] (USA)	Single	Mar 2021	34	CXR diagnoses	35.3%	Not stated	346	Mechanically Ventilated	Unclear
Housman et al[3] (USA)	Single	Sep 2020	29	CXR diagnoses	6.9 - 24%	100%	171	Mechanically Ventilated	100%**

Wong et al[4] (USA)*	Multi (2)	Nov 2020	27	CXR diagnoses	100%	Not stated	1822	ARDS	Unclear
Chopra et al[5] (USA)*	Multi (4)	May 2021	24	Not stated	100%	Not stated	842	Critical Care (594 MV)	100%
Lemmers et al[6] (Italy)	Single	Sep 2020	23	CXR/CT	0%	100%	169	ARDS	100%
Rajdev et al[7] (USA)	Single	May 2021	21	CXR/CT	Not stated	Not stated	353	Oxygen / PPV (121 MV)	100%
Brito et al[8] (Brazil)	Single	April 2021	21	100%	33%	90.5%	4087	Hospital Inpatients	100%**
Martinelli et[9] al (UK)	Multi (16)	Sep 2020	17	CXR/CT	35.3%	Not stated	6574	Hospital Inpatients	Clinical Diagnoses
Belletti et al[10] (Italy)	Single	Feb 2021	13	38.5%	53.8%	Not stated	116	ARDS Criteria	100%
Cut et al[11] (Romania)	Single	Mar 2021	11	100%	72.7%	63.6%	1648	Hospital Inpatients	Unclear
Edwards et al[12] (USA)	Single	Nov 2020	10	CXR diagnoses	20%	90%	574	Mechanically Ventilated	100%

Talan et al[13] (Turkey)	Single	Dec 2020	7	71.4%	57.1%	57.1%	161	Critical Care (96 MV)	Unclear
Udi et al[14] (Germany)	Single	Aug 2020	5	Not stated	40%	40%	20	ARDS	100%
Eperjesiova et al[15] (USA)	Single	Jul 2020	5	Not stated	20%	80%	976	Hospital Inpatients	Unclear

Studies marked with an asterix (*) focused on identifying COVID-19 pneumothorax (PTX) rather than COVID-19 pneumomediastinum (PTM) therefore all cases were PTM/PTX overlap with likely underestimation of incidence of COVID-19 PTM. Studies marked with (**) describe ‘confirmed COVID-19 infection’ rather than SARS-CoV-2 PCR positivity.

S2

Hospitals within the POETIC consortium were a representative mix of secondary and tertiary hospitals throughout the UK including those within areas of high index of multiple deprivation. They are listed below in alphabetical order:

Addenbrooke's Hospital, Cambridge
Andover War Memorial Hospital
Barnet Hospital
Basingstoke and North Hampshire Hospital, Basingstoke
Bedford Hospital
Burnley General Teaching Hospital
Chelsea and Westminster Hospital
Glangwilli Hospital, Carmarthen
Gloucester Royal Hospital
Grange University Hospital, Newport
Great Western Hospital, Swindon
John Radcliffe Hospital
Kettering General Hospital
Lister Hospital, Stevenage
Luton & Dunstable University Hospital, Luton
Musgrove Park Hospital, Taunton
Neville Hall Hospital, Abergavenny
Newham University Hospital, London
Norfolk and Norwich University Hospital, Norwich
Northumbria Specialist Emergency Care Hospital
Nottingham City Hospital
Prince Philip Hospital, Llanelli
Prince of Wales Hospital, Bridgend
Princess Alexandria Hospital, Harlow
Queen Alexandra Hospital, Portsmouth
Queen Elizabeth University Hospital, Glasgow
Royal Berkshire Hospital, Reading
Royal Blackburn Teaching Hospital
Royal Brompton Hospital, London
Royal Cornwall Hospital, Truro
Royal Derby Hospital , Derby
Royal Devon & Exeter Hospital
Royal Free Hospital, London
Royal Glamorgan Hospital, Llantrisant
Royal Gwent Hospital, Newport
Royal Hampshire County Hospital, Winchester
Royal London Hospital
Royal Stoke University Hospital
Royal United Hospitals, Bath
Saint Bartholomew's Hospital, London
Southend University Hospital
Southport & Ormskirk District General Hospital
Stoke Mandeville Hospital

Sunderland Royal Hospital
University Hospital of Coventry and Warwickshire, Coventry
University Hospital of North Durham
University Hospital of North Tees, Stockton on Tees
University Hospital of Wales, Cardiff
Watford General Hospital
Wexham Park Hospital, Wexham
Whipps Cross Hospital, London
Wythenshawe Hospital, Manchester
Ysbyty Glan Clwyd, Rhyl

S3

Table S3a. Normalization of FiO2 received by patients. The assigned FiO2 we ascribed as received by patients on differing devices.[16,17]

Device	Flow Rate (L)	Estimated FiO2 received (%)
Nasal cannulae	1	24
Nasal cannulae	2	28
Nasal cannulae	3	32
Nasal cannulae	4	36
Nasal cannulae	5	40
Nasal cannulae	6	44
Venturi Mask ‘28%’	4-6	28
Venturi Mask ‘35%’	8-10	35
Venturi Mask ‘40%’	10-12	40
Venturi Mask ‘60%’	12-15	60
Non Rebreathe Mask with Reservoir	10	62
Non Rebreathe Mask with Reservoir	11	68
Non Rebreathe Mask with Reservoir	12	72

Non Rebreathe Mask with Reservoir	13	78
Non Rebreathe Mask with Reservoir	14	84
Non Rebreathe Mask with Reservoir	15	90

Table S3b. The estimated PEEP received by patients on HFNO. This is extrapolated from expiratory pharyngeal readings for (Table 2, male subjects with their mouth closed) Groves and Tobin 2020.[18]

**Nasal Flow (L/min)**	**0**	**10**	**20**	**30**	**40**	**50**	**60**	**70**	**80**
**PEEP (cmH20)**	0.7	1.2	2.2	3.2	4.1	4.8	5.4	5.8	6.1

Domiciliary devices which entrain room air have been widely used during the pandemic. Such devices lack an oxygen blender and could result in an unreliable fraction of inspired oxygen (FiO_2).[19] Using a series of “bench” studies the ARTP COVID Group[20] have identified that amount of oxygen delivered (FiO_2) is influenced by the amount of CPAP pressure used by the patient. Ultimately increasing CPAP pressure exerts a dilutional effect on FiO_2.[20] Aware that many subjects in our data set did not have documented FiO_2 values we derived an estimate of FiO_2 for patients based upon data from ARTP COVID Group bench studies.

Using data from the ARTP Guidance for Oxygen Utilisation document,[20] we replicated a graph representing FiO_2 for varying CPAP pressure across 4 commonly used domiciliary devices at a flow rate of 15 L/min; Resmed Lumis 100, Resmed Lumis 150, Breas Vivo2 and Resmed AS10. In the figure below mean values of FiO2 for given CPAP pressures across these devices are plotted with a non-linear regression curve of best fit derived using GraphPad Prism version 9 (adapted from ARTP Guidance for Oxygen Utilisation, 2020).[20] Using the equation of this regression curve of mean values we estimated FiO_2 values for subjects in our dataset based on the assumption of 15L/min oxygen entrainment.

Figure S3c. Normalization of maximum PEEP and FiO2 for patients on CPAP

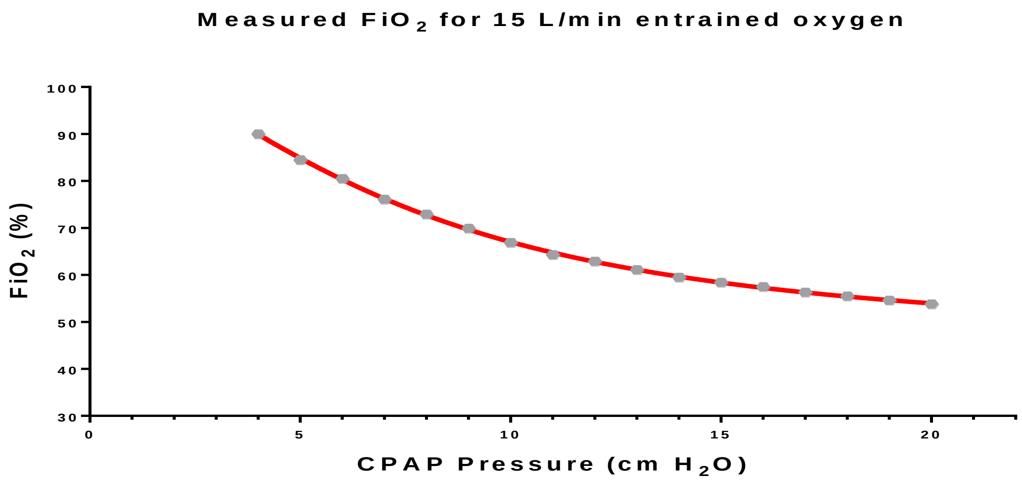

S4

Figure S4. Bubble Plot depicting the relationship between (i) the number of inpatient admissions with COVID-19 (ii) the number of these patients who had a thoracic CT scan and (iii) the number of cases of pneumomediastinum detected.*

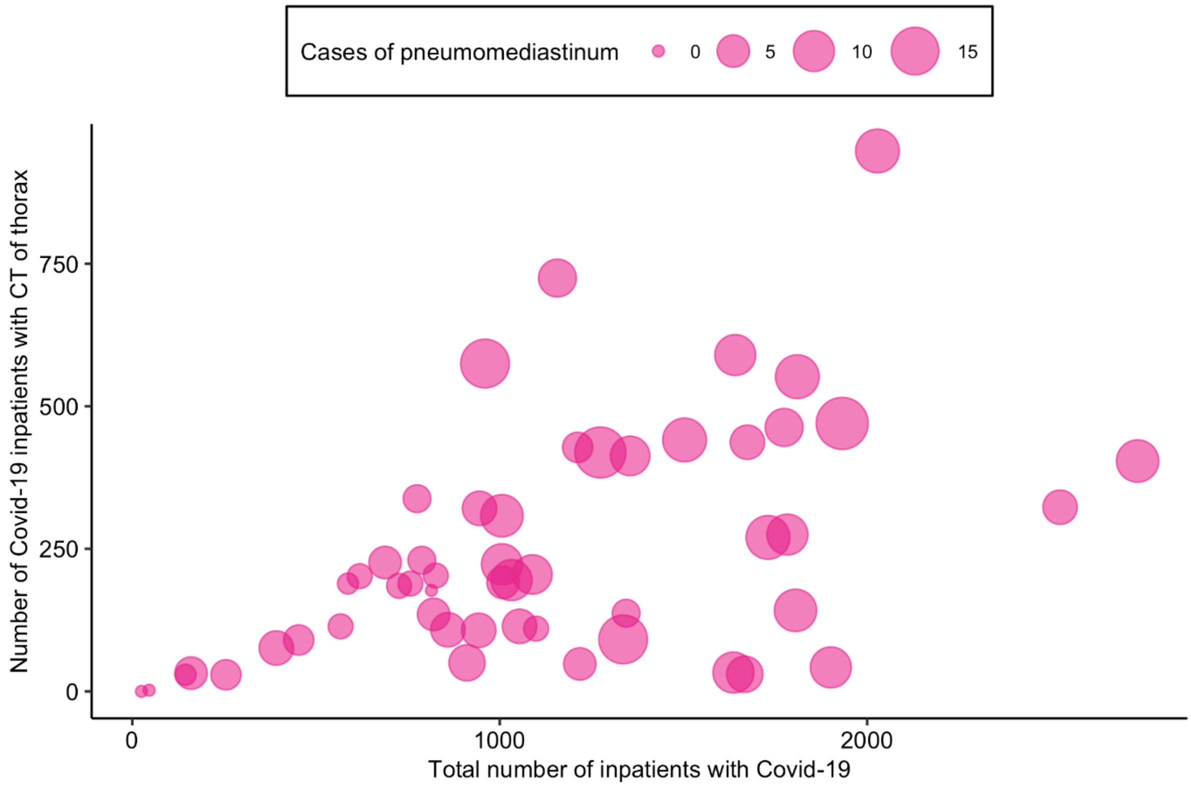

*Results from the tertiary ECMO centre the Royal Brompton Hospital are not presented within Figure S4 given the highly selective patient intake. There were 17 cases of pneumomediastinum from 87 patients admitted to the Royal Brompton Hospital with COVID-19 of whom 67 had thoracic CT scans.

Three participating hospitals had no cases of pneumomediastinum but supplied incidence data and are included. There was a strong significant correlation between the number of COVID-19 inpatients and the number of cases identified $r(50) = 0.61$ $p < .001$ and a strong correlation between the number of COVID-19 inpatients who had CT Thoraces performed and the number of cases identified $r(50) = 0.49$ $p < .001$. The number of COVID-19 inpatients having CT-scans is an inferior predictor of the number of pneumomediastinum cases than the total number of COVID-19 inpatients at a hospital (3-way ANOVA; COVID-19 inpatients $t(50) = 3.885$ $p < .001$; CT-scans $t(50) = 1.888$ $p < 0.65$).

We believe these results reflect the alternate indications for CT imaging in our COVID-19 inpatient cohort during this period which, were likely to assay for

thromboembolism and prognostication, not for pneumomediastinum. As illustrated above, there is wide variation in the amount of CT scanning in COVID-19 pneumonitis inpatients among our hospitals. This could reflect physician opinion on the utility of CT scanning in COVID-19 and possibly the varying availability of this resource at this time during the pandemic.

S5

Figure S5. Bar chart illustrating the number and frequencies of intercostal chest drains employed according to whether pneumothoraces were unilateral or bilateral (n=154). Bilateral pneumothorax was ascribed to pneumothoraces occurring on both sides of the thorax within the same admission.

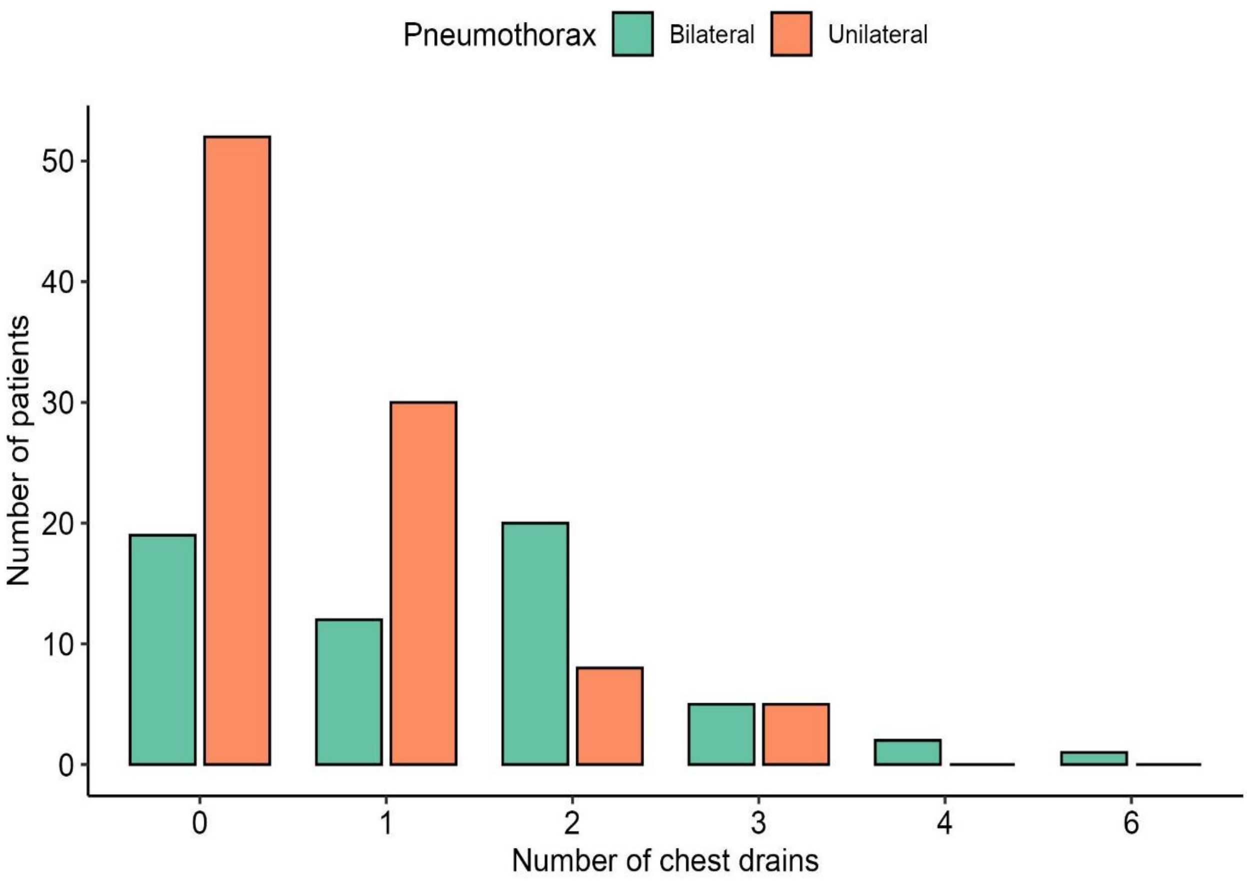

S6

Table S6a. Mortality data of patients deemed eligible for mechanical ventilation (whether mechanically ventilated or not), n = 315, Median Age = 58, (IQR 50 – 65)

	Outcome 28 Days Number (%)		Outcome 120 Days Number (%)	
**Dead**	114	(36.2)	156	(49.5)
**Discharged Home**	55	(17.5)	136	(43.2)
**Discharged Rehab**	1	(0.3)	16	(5.1)
**Still Inpatient**	145	(46.0)	7	(0.2)
	**Number of Patients (%)**			
**Received MV anytime**	241	(76.5)		
**Received ECMO**	24	(10.0)		
	**Median and IQR (Days)**			
**Length of Admission**	26	(16 – 47)		
**Duration of MV (dead)**	14	(9 – 22)		
**Duration of MV (discharged)**	24	(14 – 38)		

Table S6b. Mortality data for patients limited to CPAP support, n = 62, Median Age = 72, (IQR 61.8 – 79.3)

	Outcome 28 Days Number (%)		Outcome 120 Days Number (%)	
**Dead**	32	(51.6)	39	(62.9)
**Discharged Home**	15	(24.2)	23	(37.1)
**Discharged Rehab**	0		0	
**Still Inpatient**	15	(24.2)	0	
**Median Length of Admission**	18	(13 – 29)		

S7

A binary logistic regression model of factors predictive of death at 120 days constructed for patients for full escalation *from the point of diagnosis of pneumomediastinum* is presented in Table 2 of the main manuscript. A similar model examining the predictive utility of variables *over the course of admission* is presented below. Presenting this model separately is done (i) to include *radiographic progression* in predictive modelling as this variable is not available at the point of diagnosis and (ii) to demonstrate the dominance of mechanical ventilation as a predictor of mortality when considered across patients' admissions.

Table S7. Binary Logistic Regression Model of factors most predictive of death at 120 days *over the course of admission* for patients eligible for mechanical ventilation (n=315). All variables significantly associated with mortality in univariate analyses were entered into the model stepwise, backwards. The model produces prediction accuracy for outcome of 75.2% versus a 51.1% default accuracy.

	**B (SE)**		**Odds Ratio or % increase per unit with 95% CI**		**p**
**Mechanical ventilation**	5.01	(1.03)	150.3	(20.1 - 1125.0)	< .001
**Age**	-0.54	(0.14)	5.3 % per year	(2.7 – 7.8)	< .001
**Diabetes mellitus**	0.71	(0.38)	2.0	(1.0 – 4.3)	0.06

Variable entry into model stepwise backward. Model R^2 = 0.484 Nagelkerke χ^2 (4) = 142.1 p < .001. Constant B(SE) = 1.46 (0.88) Variables in the regression model but not listed, Radiographic Progression (p = .103). Variables not included in the model hypertension, IHD/LVSD and subcutaneous emphysema

S8

62 patients whose treatment was limited to CPAP support were analyzed separately with respect to factors associated with outcome. The following four factors were associated with increased risk of death at 120 days in univariate analysis for this group: HFNO (OR in favor of death versus oxygen 22.2, 95% CI 1.2 – 408.1, p = .04); CPAP (OR versus oxygen 4.2 , 95% CI 1.0 – 17.7, p = .05); IHD/LVSD (OR 9.8, 95% CI 1.2 – 81.2, p = 0.03) and subcutaneous emphysema (OR 3.8, 95% CI 1.3 – 11.3, p = 0.02). Notably, increasing age was *not* one of the variables associated with increased risk of death in this older subgroup. The four significantly associated variables in the univariate analyses (listed above) were entered into the regression model below.

Table S8. Binary Logistic Regression Model of factors most predictive of death at 120 days *over the course of admission* for patients whose treatment was limited to CPAP support. (n = 62). This model produces prediction accuracy for outcome of 78.3% versus a 65.0% default accuracy.

	**B (SE)**		**Odds Ratio or % increase per unit with 95% CI**		**p**
**Subcutaneous emphysema**	1.66	(0.74)	5.2	(1.2 – 22.2)	0.03

Variable entry into model stepwise backward. $R^2 = 0.533$ Nagelkerke. Model χ^2 (1) = 29.36, p < .001. Constant B(SE) = - 0.23 (0.47) Variables in the regression but not listed CPAP (p = .09). Variables not included in the model HFNO, IHD/LVSD.

S9

Figures 9a and 9b. Axial and coronal slice of thoracic CT of a mechanically ventilated patient from the cohort with COVID-19 pneumonitis, pneumomediastinum and massive subcutaneous emphysema

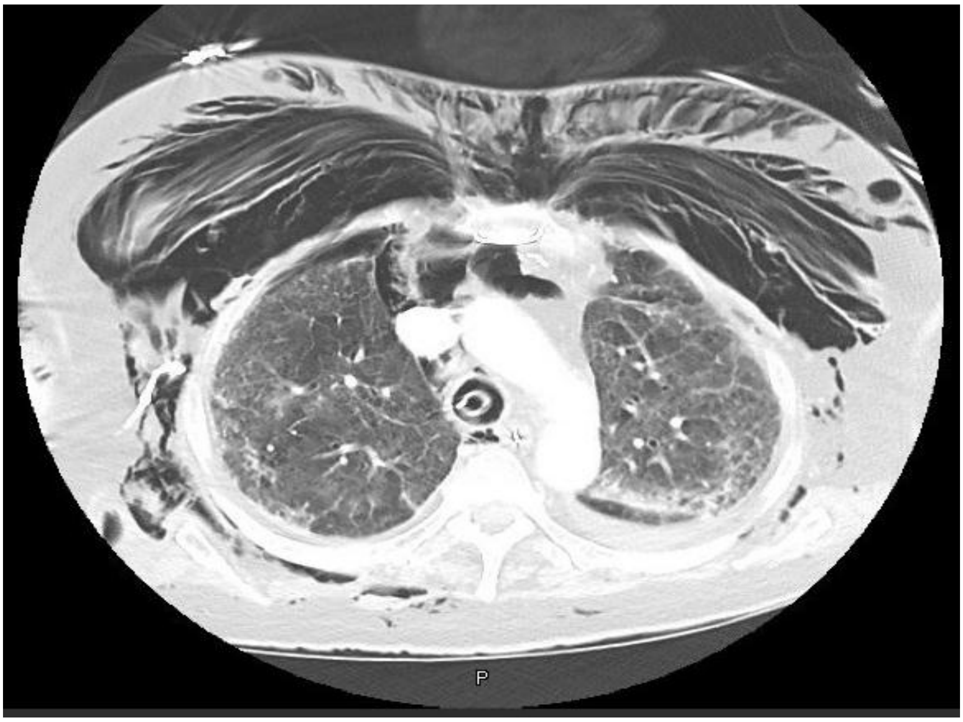

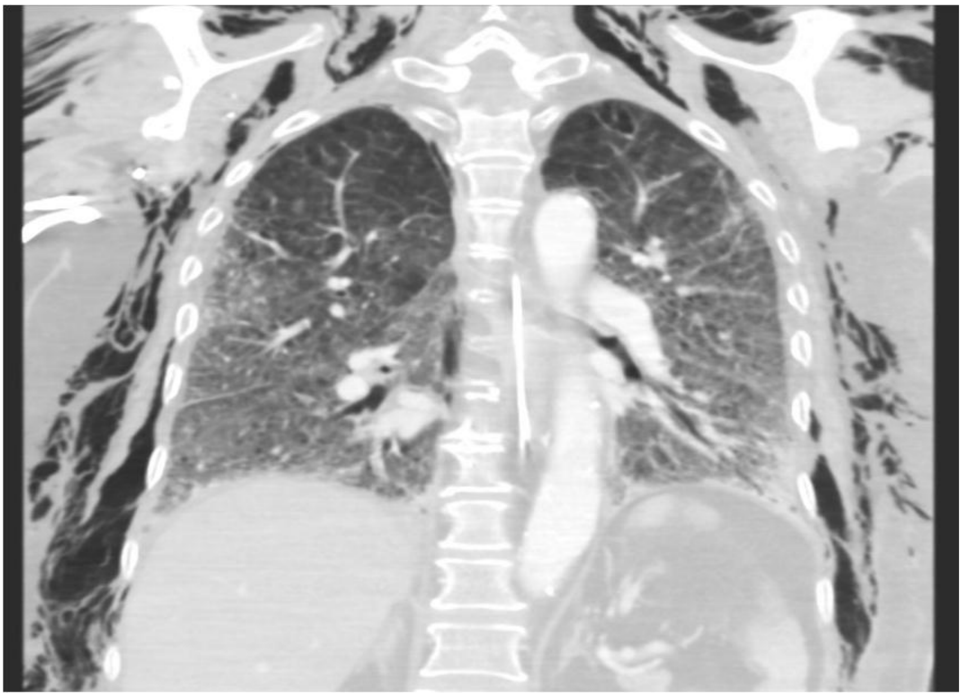

References

1. McGuinness G, Zhan C, Rosenberg N, et al. Increased Incidence of Barotrauma in Patients with COVID-19 on Invasive Mechanical Ventilation. Radiology 2020;297:E252-E62.
2. Kangas-Dick A, Gazivoda V, Ibrahim M, et al. Clinical Characteristics and Outcome of Pneumomediastinum in Patients with COVID-19 Pneumonia. J Laparoendosc Adv Surg Tech A 2021;31:273-8.
3. Housman B, Jacobi A, Carollo A, et al. COVID-19 ventilator barotrauma management: less is more. Ann Transl Med 2020;8:1575.
4. Wong K, Kim DH, Iakovou A, et al. Pneumothorax in COVID-19 Acute Respiratory Distress Syndrome: Case Series. Cureus 2020;12:e11749.
5. Chopra A, Al-Tarbsheh AH, Shah NJ, et al. Pneumothorax in critically ill patients with COVID-19 infection: Incidence, clinical characteristics and outcomes in a case control multicenter study. Respiratory Medicine 2021;184.
6. Lemmers DHL, Abu Hilal M, Bnà C, et al. Pneumomediastinum and subcutaneous emphysema in COVID-19: barotrauma or lung frailty? ERJ Open Res 2020;6.
7. Rajdev K, Spanel AJ, McMillan S, et al. Pulmonary Barotrauma in COVID-19 Patients With ARDS on Invasive and Non-Invasive Positive Pressure Ventilation. J Intensive Care Med 2021:8850666211019719.
8. Brito J, Gregório P, Mariani A, et al. Pneumomediastinum in COVID-19 disease: Outcomes and relation to the Macklin effect. Asian Cardiovasc Thorac Ann 2021;29:541-8.
9. Martinelli AW, Ingle T, Newman J, et al. COVID-19 and pneumothorax: a multicentre retrospective case series. Eur Respir J 2020;56.
10. Belletti A, Palumbo D, Zangrillo A, et al. Predictors of Pneumothorax/Pneumomediastinum in Mechanically Ventilated COVID-19 Patients. J Cardiothorac Vasc Anesth 2021.
11. Cut TG, Tudoran C, Lazureanu VE, Marinescu AR, Dumache R, Tudoran M. Spontaneous Pneumomediastinum, Pneumothorax, Pneumopericardium and Subcutaneous Emphysema-Not So Uncommon Complications in Patients with COVID-19 Pulmonary Infection-A Series of Cases. J Clin Med 2021;10.
12. Edwards JA, Breitman I, Bienstock J, et al. Pulmonary barotrauma in mechanically ventilated coronavirus disease 2019 patients: A case series. Ann Med Surg (Lond) 2021;61:24-9.
13. Talan L, Şaşal Solmaz FG, Ercan U, et al. COVID-19 pneumonia and pneumothorax: case series. Tuberk Toraks 2020;68:437-43.
14. Udi J, Lang CN, Zotzmann V, et al. Incidence of Barotrauma in Patients With COVID-19 Pneumonia During Prolonged Invasive Mechanical Ventilation - A Case-Control Study. J Intensive Care Med 2021;36:477-83.
15. Eperjesiova B, Hart E, Shokr M, Sinha P, Ferguson GT. Spontaneous Pneumomediastinum/Pneumothorax in Patients With COVID-19. Cureus 2020;12:e8996.
16. Farias E, Rudski L, Zidulka A. Delivery of high inspired oxygen by face mask. Journal of Critical Care 1991;6:119-24.
17. Whittle JS, Pavlov I, Sacchetti AD, Atwood C, Rosenberg MS. Respiratory support for adult patients with COVID-19. J Am Coll Emerg Physicians Open 2020;1:95-101.

18. Groves N, Tobin A. High flow nasal oxygen generates positive airway pressure in adult volunteers. Aust Crit Care 2007;20:126-31.
19. Messer B, Tedd H, Doris T, Mountain A, Gatilogo C, Sovani M. The variation of FiO2 with circuit type and peak inspiratory flow rate during non-invasive respiratory support using domiciliary ventilators and its significance during the COVID-19 pandemic. Journal of the Intensive Care Society;0:1751143720980280.
20. ARTP. ARTP Guidance for Oxygen Utilisation. Association of Respiratory Technology and Physiology 2020.
21. Brunson JC & Read QD (2020). “ggalluvial: Alluvial Plots in 'ggplot2'.” R package version 0.12.3, http://corybrunson.github.io/ggalluvial/.
22. Larsson J (2021). eulerr: “Area-Proportional Euler and Venn Diagrams with Ellipses.” R package version 6.1.1, https://cran.r-project.org/package=eulerr.
23. Wickham H, Averick M, Bryan J, Chang W, McGowan LD, François R, Grolemund G, Hayes A, Henry L, Hester J, Kuhn M, Pedersen TL, Miller E, Bache SM, Müller K, Ooms J, Robinson D, Seidel DP, Spinu V, Takahashi K, Vaughan D, Wilke C, Woo K, Yutani H. Welcome to the tidyverse. Journal of Open Source Software 2019;4:1686.
