# Supplementary material for: Pneumomediastinum in COVID-19: a phenotype of severe COVID-19 pneumonitis? The results of the UK POETIC survey
Source: Eur Respir J. 2022 Sep 1;60(3):2102522. doi: 10.1183/13993003.02522-2021 (PMC8832377; doi:10.1183/13993003.02522-2021)
Supplement: Supplementary file 2 [file ERJ-02522-2021.Shareable.pdf]

EUROPEAN RESPIRATORY JOURNAL
ORIGINAL RESEARCH ARTICLE
J. MELHORN ET AL.

SHAREABLE PDF

Pneumomediastinum in COVID-19: a phenotype of severe COVID-19 pneumonitis? The results of the UK POETIC survey

James Melhorn[1,2,3], Andrew Achaiah[3,4], Francesca M. Conway[5], Elizabeth M.F. Thompson[6], Erik W. Skyllberg[7], Joseph Durrant[7], Neda A. Hasan[8], Yasser Madani[8], Prasheena Naran[9], Bavithra Vijayakumar[5,10], Matthew J. Tate[11], Gareth E. Trevelyan[12], Irfan Zaki[12], Catherine A. Doig[13], Geraldine Lynch[14], Gill Warwick[15], Avinash Aujayeb[16], Karl A. Jackson[16], Hina Iftikhar[17], Jonathan H. Noble[17], Anthony Y.K.C. Ng[18], Mark Nugent[19], Philip J. Evans[19], Robert A. Hastings[20], Harry R. Bellenberg[20], Hannah Lawrence[21], Rachel L. Saville[21], Nikolas T. Johl[22], Adam N. Grey[22], Huw C. Ellis[23], Cheng Chen[23], Thomas L. Jones[24], Nadeem Maddekar[25], Shahul Leyakathali Khan[25], Ambreen Iqbal Muhammad[26], Hakim Ghani[26], Yadee Maung Maung Myint[26], Cecillia Rafique[27], Benjamin J. Pippard[27], Benjamin R.H. Irving[28], Fawad Ali[29], Viola H. Asimba[30], Aqeem Azam[31], Eleanor C. Barton[32], Malvika Bhatnagar[33], Matthew P. Blackburn[34], Kate J. Millington[35], Nicholas J. Budhram[35], Katherine L. Bunclark[36], Toshit P. Sapkal[36], Giles Dixon[37], Andrew J.E. Harries[38], Mohammad Ijaz[39], Vijayalakshmi Karunanithi[40], Samir Naik[40], Malik Aamaz Khan[41], Karishma Savlani[41], Vimal Kumar[42], Beatriz Lara Gallego[43], Noor A. Mahdi[44], Caitlin Morgan[45], Neena Patel[46], Elen W. Rowlands[47], Matthew S. Steward[48], Richard S. Thorley[49], Rebecca L. Wollerton[49], Sana Ullah[50], David M. Smith[51], Wojciech Lason[1], Anthony J. Rostron[51], Najib M. Rahman[2,52] and Rob J. Hallifax[1,3]

[1]Nuffield Dept of Medicine, John Radcliffe Hospital, University of Oxford, Oxford, UK. [2]National Institute for Health Research (NIHR) Oxford Biomedical Research Centre, Oxford, UK. [3]John Radcliffe Hospital, Oxford University Hospitals NHS Foundation Trust, Oxford, UK. [4]MRC Human Immunology Unit, Weatherall Institute of Molecular Medicine, University of Oxford, Oxford, UK. [5]Royal Brompton Hospital, National Heart and Lung Institute, London, UK. [6]St Bartholomew's Hospital, Barts Health NHS Trust, London, UK. [7]Newham University Hospital, Barts Health NHS Trust, London, UK. [8]Wexham Park Hospital, Frimley Health NHS Foundation Trust, Slough, UK. [9]Royal Free Hospital, Royal Free London NHS Foundation Trust, London, UK. [10]Chelsea and Westminster Hospital, National Heart and Lung Institute, Imperial College London, London, UK. [11]Queen Elizabeth University Hospital, NHS Greater Glasgow and Clyde, Glasgow, UK. [12]Royal Berkshire Hospital, Royal Berkshire NHS Foundation Trust, Reading, UK. [13]Southend University Hospital, Mid and South Essex NHS Foundation Trust, Southend, UK. [14]Prince of Wales Hospital, Cwm Taf Morgannwg University Health Board, Bridgend, UK. [15]The Royal Gwent Hospital, Aneurin Bevan Health Board, Newport, UK. [16]Northumbria Specialist Emergency Care Hospital, Northumbria Healthcare NHS Foundation Trust, Cramlington, UK. [17]Gloucester Royal Hospital, Gloucestershire Hospitals NHS Foundation Trust, Gloucester, UK. [18]Addenbrookes Hospital, Cambridge University Hospitals NHS Trust, Cambridge, UK. [19]Glangwilli General Hospital, Hywel Dda University Health Board, Carmarthen, UK. [20]Barnet General Hospital, Royal Free London NHS Foundation Trust, London, UK. [21]Royal Derby Hospital, University Hospitals of Derby and Burton NHS Foundation Trust, Derby, UK. [22]University Hospital of Wales, Cardiff and Vale University Health Board, Cardiff, UK. [23]Stoke Mandeville Hospital, Buckinghamshire Healthcare NHS Trust, Stoke Mandeville, UK. [24]Basingstoke and North Hampshire Hospital, Hampshire Hospitals NHS Foundation Trust, Basingstoke, UK. [25]Royal Stoke University Hospital, University Hospitals of North Midlands NHS Trust, Stoke, UK. [26]Watford General Hospital, West Hertfordshire Hospitals NHS Trust, Watford, UK. [27]University Hospital of North Tees, North Tees and Hartlepool NHS Foundation Trust, Hartlepool, UK. [28]Queen Alexandra Hospital, Portsmouth Hospitals University NHS Trust, Portsmouth, UK. [29]Bedford Hospital, Bedfordshire Hospitals NHS Foundation Trust, Bedford, UK. [30]Nottingham University Hospital, Nottingham University Hospitals NHS Trust, Nottingham, UK. [31]Royal Blackburn Teaching Hospital, East Lancashire Hospitals NHS Trust, Blackburn, UK. [32]The Grange University Hospital, Aneurin Bevan Health Board, Cwmbran, UK. [33]Darlington Memorial Hospital, County Durham and Darlington NHS Foundation Trust, Durham, UK. [34]Southport and Ormskirk District General Hospital, Southport and Ormskirk Hospital NHS Trust, Southport, UK. [35]Great Western Hospital, Great Western Hospital NHS Foundation Trust, Swindon, UK. [36]Norwich and Norfolk University Hospital, Norfolk and Norwich University Hospitals NHS Foundation Trust, Norwich, UK. [37]Royal United Hospitals Bath, The Royal United Hospitals Bath NHS Foundation Trust, Bath, UK. [38]Royal Glamorgan Hospital, Llantrisant, Cwm Taf University Health Board, Llantrisant, UK. [39]Wythenshawe Hospital, Manchester University NHS Foundation Trust, Manchester, UK. [40]The Princess Alexandra Hospital, The Princess Alexandra Hospital NHS Trust, Harlow, UK. [41]Luton and Dunstable University Hospital, Bedfordshire Hospitals NHS Foundation Trust, Luton, UK. [42]Kettering General Hospital, Kettering General Hospital NHS Foundation Trust, Kettering, UK. [43]University Hospital, University Hospitals Coventry and Warwickshire NHS Trust, Coventry, UK. [44]Lister Hospital, East and North Hertfordshire NHS Trust Stevenage, Stevenage, UK. [45]Musgrove Park Hospital, Somerset NHS Foundation Trust, Taunton, UK. [46]Whipps Cross Hospital, Barts Health NHS Trust, London, UK. [47]Neville Hall Hospital, Aneurin Bevan University Health Board, Abergavenny, UK. [48]Royal Devon and Exeter Hospital, Royal Devon and Exeter NHS Foundation Trust, Exeter, UK. [49]The Royal Cornwall Hospital, Royal Cornwall Hospitals NHS Trust, Truro, UK. [50]Ysbyty Glan Clwyd Hospital, Betsi Cadwaladr University Health Board, Rhyl, UK. [51]Integrated Critical Care Unit, Sunderland Royal Hospital, South Tyneside and Sunderland NHS Foundation Trust, Sunderland, UK. [52]Oxford Centre for Respiratory Medicine, Nuffield Dept of Medicine, University of Oxford, Oxford, UK.

Corresponding author: James Melhorn (james.melhorn@ndm.ox.ac.uk)

Shareable abstract (@ERSpublications)
Roughly 0.6% of patients admitted with COVID-19 have pneumomediastinum identified. The finding is associated with severe COVID-19 and high mortality. https://bit.ly/3nXlaR5

Cite this article as: Melhorn J, Achaiah A, Conway FM, *et al*. Pneumomediastinum in COVID-19: a phenotype of severe COVID-19 pneumonitis? The results of the UK POETIC survey. *Eur Respir J* 2022; 60: 2102522 [DOI: 10.1183/13993003.02522-2021].

This single-page version can be shared freely online.

Copyright ©The authors 2022.

This version is distributed under the terms of the Creative Commons Attribution Licence 4.0.

Received: 19 Sept 2021
Accepted: 12 Jan 2022

Abstract

Background There is an emerging understanding that coronavirus disease 2019 (COVID-19) is associated with increased incidence of pneumomediastinum (PTM). We aimed to determine its incidence among patients hospitalised with COVID-19 in the UK and describe factors associated with outcome.

Methods A structured survey of PTM and its incidence was conducted from September 2020 to February 2021. UK-wide participation was solicited *via* respiratory research networks. Identified patients had severe acute respiratory syndrome coronavirus 2 (SARS-CoV-2) infection and radiologically proven PTM. The primary outcomes were to determine incidence of PTM in COVID-19 and to investigate risk factors associated with patient mortality.

Results 377 cases of PTM in COVID-19 were identified from 58 484 inpatients with COVID-19 at 53 hospitals during the study period, giving an incidence of 0.64%. Overall 120-day mortality in COVID-19 PTM was 195 out of 377 (51.7%). PTM in COVID-19 was associated with high rates of mechanical ventilation. 172 out of 377 patients (45.6%) were mechanically ventilated at the point of diagnosis. Mechanical ventilation was the most important predictor of mortality in COVID-19 PTM at the time of diagnosis and thereafter ($p<0.001$), along with increasing age ($p<0.01$) and diabetes mellitus ($p=0.08$). Switching patients from continuous positive airway pressure support to oxygen or high-flow nasal oxygen after the diagnosis of PTM was not associated with difference in mortality.

Conclusions PTM appears to be a marker of severe COVID-19 pneumonitis. The majority of patients in whom PTM was identified had not been mechanically ventilated at the point of diagnosis.
